# Efectividad de las intervenciones para revertir el diagnóstico del síndrome metabólico: actualización de un metaanálisis de comparación mixta de tratamientos

**DOI:** 10.7705/biomedica.4684

**Published:** 2019-12-30

**Authors:** Adriana Guzmán, Esteban Navarro, Leidy Obando, Jorge Pacheco, Korina Quirós, Leticia Vásquez, Milena Castro, Fernando Ramírez

**Affiliations:** 1 Escuela de Estadística, Facultad de Ciencias Económicas, Universidad de Costa Rica, San José, Costa Rica Universidad de Costa Rica Escuela de Estadística Facultad de Ciencias Económicas Universidad de Costa Rica San José Costa Rica; 2 Centro de Investigación en Matemática Pura y Aplicada, Universidad de Costa Rica, San José, Costa Rica Universidad de Costa Rica Centro de Investigación en Matemática Pura y Aplicada Universidad de Costa Rica San José Costa Rica

**Keywords:** síndrome metabólico, diabetes mellitus de tipo 2, enfermedades cardiovasculares, metaanálisis, oportunidad relativa., Metabolic syndrome, diabetes mellitus, type 2, cardiovascular diseases, metaanalysis, odds ratio

## Abstract

**Introducción.:**

El conocer las intervenciones más efectivas para revertir el síndrome metabólico es clave para el diseño de estrategias clínicas de prevención de enfermedades como la diabetes mellitus de tipo 2 y la enfermedad cardiovascular.

**Objetivo.:**

Sintetizar el tamaño del efecto de las intervenciones disponibles para revertir un diagnóstico de síndrome metabólico.

**Materiales y métodos.:**

Se hizo la búsqueda en Embase y Medline, incluyendo los ensayos clínicos en los que la variable “respuesta” se definía como la reversión del diagnóstico del síndrome metabólico. Se categorizaron las intervenciones en cuatro dimensiones: 1) estilo de vida (dieta y ejercicio); 2) farmacia; 3) combinación de estilo de vida y farmacia, y 4) grupos de control; finalmente, se hizo una comparación mixta de tratamientos.

**Resultados.:**

Se detectaron dos estudios adicionales a los incluidos en el metaanálisis publicado por Dunkley, et al., en el 2012. Se estimó que las intervenciones relacionadas con el estilo de vida tuvieron 2,61 veces (intervalo de credibilidad entre 1,00 y 5,47) más probabilidades de revertir el síndrome metabólico que las de los grupos de control y las relacionadas con los tratamientos farmacéuticos, una probabilidad de 3,39 veces más que las del grupo de control, pero con un intervalo de credibilidad entre 0,81 y 9,99. Las intervenciones sobre el estilo de vida tuvieron 1,59 veces más probabilidades de revertir el síndrome metabólico que las del tratamiento farmacéutico.

**Conclusión.:**

Las estrategias basadas en la dieta y la actividad física de las personas, tuvieron una mayor probabilidad de ser más efectivas para revertir el diagnóstico de síndrome metabólico.

## Abstract (en)

**Introduction:**

Identifying the most effective interventions to reverse the metabolic syndrome can be key in the design of clinical strategies to prevent progression to type 2 diabetes mellitus and cardiovascular disease.

**Objective:**

To estimate the effect size of the interventions used for the reversal of metabolic syndrome.

**Materials and methods:**

We searched in Embase and Medline databases for randomized clinical trials with an outcome defined as the reversal of the metabolic syndrome diagnosis. We classified the interventions in four dimensions: 1) lifestyle (diet and exercise); 2) pharmaceuticals; 3) a combination of both, and 4) control groups, and we conducted a mixed treatment comparison analysis.

**Results:**

Additional to the previous meta-analysis published by Dunkley, et al. in 2012, we identified two other studies. Lifestyle interventions had 2.61 more chances to achieve the reversal of the metabolic syndrome than the control group, with a credible interval between 1.00 and 5.47. Pharmaceutical treatments showed a 3.39 higher chance of reversing the syndrome compared with the control group, but the credible interval was estimated from 0.81 to 9.99. Lifestyle interventions had 1.59 more chance of reversal than the pharmaceutical treatments.

**Conclusion:**

Diet and physical activity-based interventions had a higher probability of effectiveness to reverse a metabolic syndrome diagnosis.

Los estudios sobre el síndrome metabólico adoptan las definiciones planteadas por la Organización Mundial de la Salud (OMS), la *International Diabetes Federation* (IDF) y el *National Cholesterol Education Program Adult Treatment Panel III* (NCEP-ATP III) (NCEP). Según este último, el síndrome metabólico se define como la agrupación, por lo menos, de tres factores de riesgo cardiovascular de una lista de cinco (glucosa elevada en plasma en ayuno, presión arterial elevada, triglicéridos elevados, niveles bajos de HDL y obesidad abdominal según los valores de la circunferencia de la cintura), en tanto que la elevación de la insulina constituye un factor relevante en la definición de la OMS y la obesidad abdominal es el factor principal para la IDF ^1^.

Las estimaciones de la prevalencia del síndrome metabólico se ven influenciadas directamente por el tipo de definición utilizada y las variaciones que se presentan al medir la obesidad; por ejemplo, en Francia se reporta una prevalencia de 10 % en hombres y de 7 % en mujeres con edades entre los 30 y los 64 años al emplear los parámetros del NCEP y, de 23,5 %, aproximadamente, en hombres y alrededor del 9,2 % en mujeres en el mismo rango de edad, al emplear los de la OMS ^2^.

La divergencia según las definiciones introduce una considerable heterogeneidad en la comparación de la prevalencia a nivel mundial ^3^. Sin embargo, con la definición del NCEP hay estudios en países como México, con el 26,6 % para hombres y mujeres entre los 20 y los 69 años de edad ^4^; en Irlanda, se ha estimado una prevalencia del síndrome metabólico de 21,8 % para hombres y de 21,5 % para mujeres entre los 50 y los 69 años de edad ^5^; en Estados Unidos, se ha estimado una prevalencia de 24,2 % en hombres y de 23,5 % en personas mayores de 19 años ^6^, y en India, de 7,9 % en hombres y de 17,5 % en mujeres mayores de 20 años ^7^, entre otros ^3^.

La investigación sobre el síndrome metabólico se ha tornado crucial para la prevención de la diabetes de tipo 2 ^8^, por lo que el uso de una definición que no considere los altos niveles de insulina es crucial en el análisis detallado del fenómeno y para proponer estrategias de salud pública. Asimismo, este síndrome está asociado con un riesgo moderado de desarrollar enfermedades cardiovasculares ^9^^,^^10^ y con un riesgo considerable de desarrollar diabetes de tipo 2 ^11^; según la actualización de las revisiones llevada a cabo por una de las autoras (en proceso de publicación), se estima que el riesgo relativo de mortalidad por enfermedad cardiovascular en personas con síndrome metabólico es 1,67 veces mayor que en las personas que no lo tienen y, para la diabetes de tipo 2, el riesgo relativo se incrementa hasta 4,31 veces en personas con el síndrome.

En este contexto, el estudio de dicho síndrome resulta de gran relevancia por su relación con la obesidad como factor de riesgo. La detección de los individuos en la práctica clínica es crucial para el planteamiento de políticas públicas de salud a nivel mundial y tiene una gran posibilidad de incidir en la reducción de la prevalencia de la diabetes de tipo 2, los accidentes cerebrovasculares y las enfermedades del corazón en general, tanto a nivel de los incidentes específicos, como de la mortalidad general. 

Por lo tanto, el determinar las intervenciones más efectivas es un requisito en los procesos de investigación. En la revisión sistemática de Dunkley, et al. ^12^, en el 2012, se hizo una primera síntesis de los datos disponibles mediante un metaanálisis de comparaciones mixtas de tratamientos, la cual resulta pertinente, dado que no todas las intervenciones disponibles se han contrastado en forma directa ^13^. Los tratamientos propuestos para el síndrome metabólico se pueden agrupar en estrategias relacionadas con el estilo de vida, con el tratamiento farmacológico o con su combinación. En el estudio de Dunkley, *et al*., no se establece qué tipo de tratamiento sobresale de forma singular.

En el presente artículo, se presenta una actualización de la evidencia científica que permita especificar mejor la intervención con mayores probabilidades de ser efectiva.

## Materiales y métodos

Se recolectaron los datos secundarios de las publicaciones disponibles cuya pregunta de investigación se hubiera centrado en la observación del tamaño del efecto de las intervenciones y sus diferentes aproximaciones tecnológicas, con el fin de recomendarlas en el marco de las políticas públicas en salud.

Se hizo una revisión sistemática para extraer los datos requeridos y establecer un contraste entre las diferentes opciones tecnológicas, basadas en esquemas de dieta y ejercicio, y de tratamientos farmacológicos ^14^. Este tipo de datos posibilita el estudio de un parámetro de medición, en este caso, la razón de probabilidades o momios (*odds ratio*, OR) y la utilización de métodos de síntesis de la evidencia para estimar este tipo de medidas ofrece mayor precisión frente a la heterogeneidad de las poblaciones.

Por lo tanto, se parte de la necesidad de determinar la mejor intervención contra el síndrome metabólico para, así, prevenir el avance acelerado hacia un mayor compromiso de la salud, como la enfermedad cardiovascular o la diabetes mellitus de tipo 2.

La revisión comprendió intervenciones incluidas en alguna de las siguientes cuatro categorías: control, intervenciones sobre el estilo de vida (dieta o ejercicio), tratamientos farmacológicos o la combinación de estas dos últimas. En este sentido, se hizo una búsqueda exhaustiva de la ‘evidencia’ científica publicada en artículos sobre estudios clínicos cuyos resultados incluyeran la proporción de las personas en cada uno de los grupos de intervención en quienes hubo reversión del síndrome metabólico.

Se siguieron rigurosas estrategias de validación de los datos requeridos en cada etapa del proceso, con el fin de garantizar la calidad de los datos extraídos y analizados.

### Estrategia de búsqueda

En la búsqueda se utilizaron palabras clave en tres categorías: tipo de población representada en la muestra, características de interés relacionadas con el síndrome metabólico y especificaciones metodológicas para la detección de estudios clínicos.

Se definió cada conjunto a partir de la unión de las palabras clave de cada una de las categorías. La población se configuró a partir de las palabras adult, aged, female, male y human. En cuanto a las características de base de cada estudio, se recopilaron los términos *metabolic syndrome, metabolic syndrome X, glucose, triglycerides, hypertension, obesity, diabetes mellitus, cardiovascular disease, cardiovascular risk y metabolic cardiovascular syndrome*. Para recopilar el conjunto de estudios clínicos, se utilizaron los términos *article, priority journal, clinical trial, controlled clinical trial, controlled study, randomized controlled trial, randomized trial y placebo*.

Después de definir los conjuntos, se diseñó la estrategia para obtener la intersección de las tres categorías, y los estudios resultantes se clasificaron según los criterios de inclusión y exclusión para obtener aquellos que conformaran la base analítica para determinar la mejor intervención de reversión del síndrome metabólico.

La estrategia de búsqueda se validó con los artículos incluidos en el estudio de base ^12^, cuyas palabras clave se extrajeron para calcular su frecuencia, con el propósito de incluir solo aquellas que estuvieran presentes, por lo menos, en cinco artículos.

En la segunda etapa, se implementó la estrategia de búsqueda en las bases de datos. Para los documentos en lengua extranjera, se hicieron traducciones parciales o completas para comprobar el cumplimiento de los criterios de inclusión.

La búsqueda se hizo en las bases de datos referenciales de Embase y Medline. La actualización consistió en revisar los estudios relevantes que hubiesen sido publicados entre enero del 2010 y octubre del 2017, ya que el estudio de base había incluido los estudios anteriores a enero del 2010 ^12^. Una vez se completó la búsqueda en cada base de datos, se excluyeron los duplicados y se diseñó una base de datos para registrar los documentos relevantes para la revisión y el metaanálisis. La búsqueda se validó eliminando el filtro por año y verificando la aparición de los 13 artículos incluidos en el estudio de referencia, los cuales efectivamente se encontraron, con lo cual se validó la estrategia de búsqueda para la actualización.

**Figura 1.**
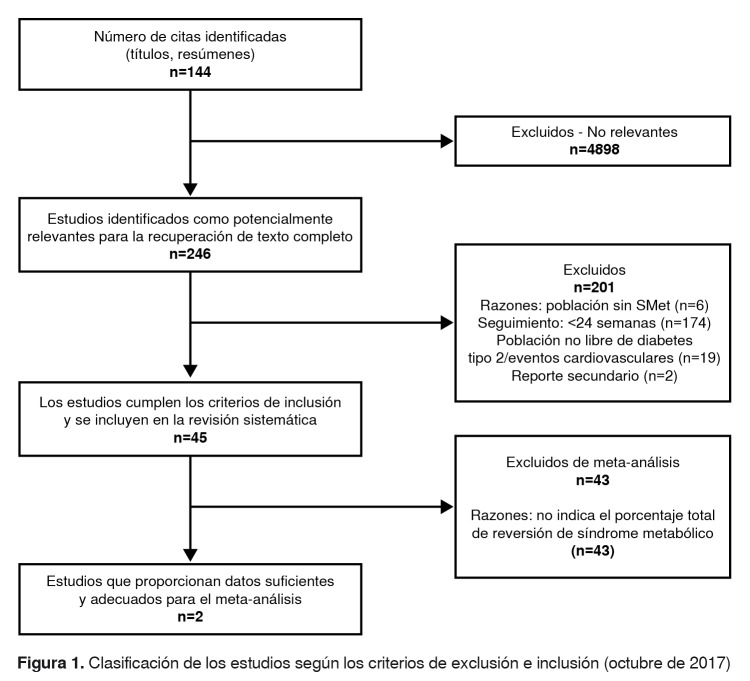
Clasificación de los estudios según los criterios de exclusión e inclusión (octubre de 2017)

Selección de los estudios La búsqueda arrojó 5.144 títulos en los cuales se usaron los criterios de inclusión y exclusión específicos para seleccionar el tipo de información requerida para comparar las diferentes opciones tecnológicas empleadas en la resolución de una condición con las características del síndrome metabólico. En la figura 1 se presenta el proceso de selección de los estudios. 

### Criterios de inclusión

Los criterios de inclusión fueron los siguientes: estudios clínicos controlados en poblaciones humanas mayores de 18 años cuya muestra incluyera una proporción de personas (mayor de 0) con diagnóstico de síndrome metabólico y un seguimiento mayor o igual a 24 semanas. Además, se consideró la inclusión, por lo menos, de dos tipos de intervención de los tres contemplados: el de intervenciones relacionadas con el estilo de vida de las personas (basadas en componentes de dieta, ejercicio o ambas), el de los tratamientos farmacéuticos disponibles, o los que combinaban estilo de vida y farmacoterapia.

Todos los estudios incluidos incorporaban, por lo menos, un nivel de comparación basal o un placebo. No hubo restricción por el idioma y solo se incluyeron aquellos en los que se daba una definición de síndrome metabólico acorde con los criterios especificados por el NCEP. 

### Criterios de exclusión

Se revisaron los resúmenes de los artículos para determinar si cumplían con los requisitos del análisis y se excluyeron los que reportaban la medición de un efecto por componente del síndrome, en lugar de un efecto del conglomerado de componentes. Se consideraron solo aquellos artículos en los que la definición de la variable ‘respuesta’ siguiera una distribución binomial, es decir, resultado positivo si la persona del grupo de intervención cumplía con los criterios del diagnóstico del síndrome metabólico, y resultado negativo si la característica de interés estaba ausente.

Se excluyeron todos los artículos que ofrecían una respuesta multivariada, y aquellos que no cumplían con los mismos criterios de inclusión y exclusión definidos por el estudio de referencia ^12^. Además, se excluyeron los documentos que no ofrecían los datos de extracción especificados para dicho metaanálisis. 

### Extracción de los datos

Se diseñó una estrategia para la extracción y la evaluación de la calidad de los datos seleccionados, utilizando las palabras clave de los trece artículos seleccionados en el estudio de base. Se emplearon las funciones condicionales en Excel, para verificar que los artículos seleccionados cumplieran con los términos y los criterios de inclusión y exclusión.

Se consideraron las variables características del estudio y el documento de publicación, el tamaño de muestra, las características demográficas de la población de interés, los tiempos de seguimiento, la proporción relativa de los registros de reversión del síndrome metabólico (porcentaje) o su conteo absoluto. En algunos casos, fue necesario hacer transformaciones para obtener los porcentajes, dado que la medida de interés se reportó como OR.

### Síntesis de la evidencia

En la búsqueda de artículos posteriores al estudio de base ^12^, se encontraron dos estudios con datos suficientes para ser incluidos en el metaanálisis. Dadas las características de la ‘evidencia’, es necesario comparar los métodos de síntesis de la información; en este caso, se evaluó un modelo asumiendo efectos fijos y efectos aleatorios no correlacionados. Asimismo, dado que la ‘evidencia’ no se presentó vinculada, es decir, no todas las comparaciones posibles entre los tratamientos se encontraban en el mismo estudio clínico, la estimación de la OR debe basarse en el cálculo de las comparaciones que no se han hecho directamente en todos los estudios.

A este tercer tipo de modelo se le denomina ‘método de comparaciones mixtas de tratamientos’, ya que se basa en estimaciones directas e indirectas según la disponibilidad de los datos ^15^^,^^16^. También se le conoce como metaanálisis de múltiple tratamiento (*Multiple Treatment Meta-Analysis*) o metaanálisis de redes (*Network Meta-Analysis*) ^13^^,^^17^. Para analizar este aspecto, se puede plantear un análisis geométrico a partir de anotar una red de información o de tratamientos. Las ventajas de este enfoque analítico es que permite estimar la probabilidad de eventos sobre los cuales se requiere tomar una decisión crítica, a pesar de la escasez de ‘evidencia’ científica.

En todos los análisis, se utilizó la comparación por pares de cada una de las intervenciones: la de control, la relacionada con el estilo de vida o con los fármacos y la combinación de estas dos. En la compilación de ambos modelos, se incorporaron solo seis de los trece artículos del estudio de base y los dos artículos detectados en la búsqueda durante el periodo adicional de observación, esto, con la finalidad de mantener la relación en la red de tratamientos durante la convergencia de los modelos, por lo que la estimación de la OR correspondió a la síntesis de las medidas reportadas en ocho de los artículos.

### Red de tratamientos

**Figura 2.**
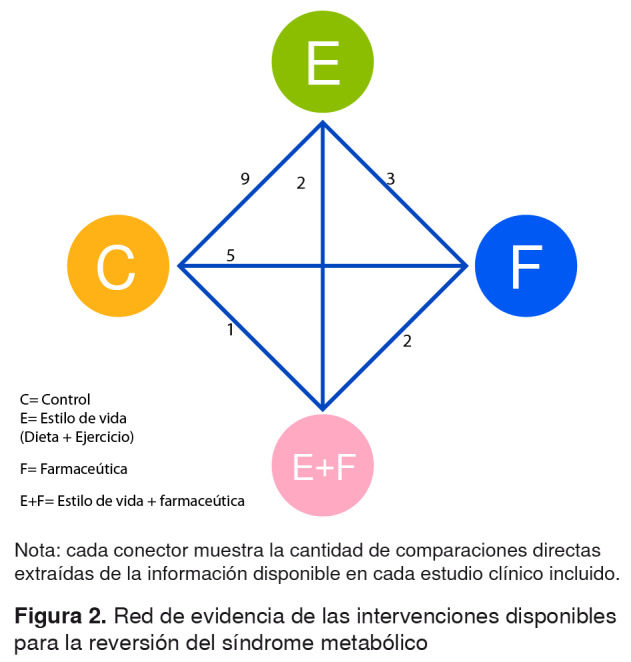
Red de evidencia de las intervenciones disponibles para la reversión del síndrome metabólico

La red construida para este estudio se presenta en la figura 2 y se basa en la integración de la evidencia en las cuatro categorías comparadas en este análisis. Cada categoría conformó un tipo de tratamiento según el enfoque tecnológico.

El control se definió como el comparador y se refiere a aquellas intervenciones de base en las que se observó la probabilidad de que el síndrome metabólico se revirtiera en una persona. Las intervenciones diseñadas para mejorar los hábitos nutricionales con la dieta, se integraron en la categoría de estilo de vida conjuntamente con las propuestas de entrenamiento físico. Los tratamientos farmacológicos podían incluir los medicamentos contra la obesidad y los antidiabéticos.

Por último, se contempló la combinación de un tratamiento farmacológico con los esquemas de estilo de vida. En la figura 2, se aprecia la distribución de la ‘evidencia’ según el número de comparaciones directas halladas en los artículos, siendo el contraste entre el control y el estilo de vida la relación con mayor evidencia directa, seguida del tratamiento farmacológico comparado con un control. El binomio con menor ‘evidencia’ fue el de un control y la combinación de las intervenciones. En este caso, la red quedó completamente conectada, lo que garantizó la solidez de los resultados analíticos.

### Análisis estadístico

La OR de cada comparación requerida se calculó utilizando un enfoque de estadística bayesiana ^18^ mediante el programa de WinBUGS en cada uno de los modelos usados: efecto fijo, efecto aleatorio y comparaciones mixtas de tratamientos para considerar la correlación de las comparaciones. La plataforma de WinBUGS utiliza un algoritmo basado en el método de Monte Carlo con cadenas de Markov (*Markov chain Monte Carlo*, MCMC) y la herramienta de muestreo de Gibbs para estimar los parámetros estadísticos (media, mediana y desviación estándar) de una función de densidad posterior ^19^^-^^22^. Cada modelo se compiló con un desecho de las primeras 5.000 iteraciones y los parámetros reportados en este artículo se basan en la estimación ofrecida por la función de densidad posterior generada luego de 10.000 iteraciones.

El análisis de los modelos se hizo en la versión 14 de la plataforma de software de WinBUGS y el diagrama de bosque con el programa R (versión 3,1), en tanto que las tablas se formatearon con Excel. En la preparación de estos resultados, se siguió la guía PRISMA ^23^.

La selección de los estudios y la extracción de los datos estuvieron a cargo de un equipo de cinco investigadores independientes y se usaron diferentes estrategias para corroborar la exhaustividad de la estrategia de búsqueda y la detección de estudios, así como la calidad de la extracción de los datos, tal como se especificaron en las secciones anteriores.

## Resultados

Se seleccionaron 45 artículos según los criterios de inclusión y exclusión. En la figura 1, se muestra la primera selección de 246 documentos. Tras revisar la prevalencia del síndrome metabólico antes de la intervención y después de ella, solamente en dos artículos ^24^^,^^25^ se utilizó un enfoque binomial para clasificar el síndrome, es decir, reportaron si cumplían o no con los criterios para su diagnóstico.

En los 15 estudios que hicieron parte de la revisión sistemática, se incluyeron los siguientes tratamientos implementados y evaluados por separado o de manera combinada: (i) asesoramiento dietético individualizado e intensivo; (ii) sesiones supervisadas de ejercicio; (iii) consejos de ejercicios; (iv) metformina; (v) rosiglitazona; (vi) atorvastatina; (vii) pravastatina; (viii) lovastatina; (ix) fenofibrato; (x) sibutramina, y (xi) rimonabant.

Se encontraron ocho estudios centrados únicamente en la efectividad de las intervenciones relacionadas con el estilo de vida (uno, solo dieta; dos, solo ejercicios; cuatro, dietas combinadas con ejercicio, y uno, dieta, educación y ejercicio). En cuatro estudios, se compararon la efectividad de las intervenciones farmacológicas y, en tres de ellos, se combinaron intervenciones farmacológicas con las relacionadas con el estilo de vida. En doce de los estudios se hizo análisis de subgrupos, y ocho de estos fueron publicados posteriormente en documentos separados (post hoc). Los artículos revisados se habían publicado en los 11 años anteriores y los estudios se llevaron a cabo en Grecia, Noruega, Italia, Holanda, Estados Unidos, China y Corea.

Se utilizó la definición del NCEP como criterio de inclusión de los estudios en el metaanálisis. Con respecto a la duración de los estudios, se tuvieron en cuenta períodos de seguimiento a partir de 24 semanas y hasta cuatro años. La edad media de los participantes y el índice de masa corporal, variaron de 44 a 70 años y de 25,6 a 39 kg/m 2 , respectivamente. El porcentaje de hombres fluctuó entre 21 y 100 %. Tal como en el estudio de base, se definió la reversión del síndrome metabólico según la proporción de individuos con diagnóstico positivo en el estado inicial del estudio y cuya condición cambió en el curso del seguimiento.

Los dos estudios informaron adecuadamente sobre los criterios de inclusión en el análisis y los porcentajes de reversión del síndrome metabólico, pero solo en uno se reportaron las OR de la reversión para el tratamiento y el correspondiente intervalo de confianza para la estimación.

Como se constata en la red de tratamientos presentada en la figura 2, en los artículos incluidos en el análisis se encontraron cuatro diferentes comparaciones entre los tratamientos. En el cuadro 1, se presentan las características de los estudios revisados, y se registran los autores y el año de publicación, el país, el tamaño de la muestra, el tipo de intervención, y las características de los participantes y del diseño del estudio, así como las definiciones utilizadas.

**Cuadro 1:** Detalles de todos los estudios incluidos en la revisión sistemática y descripción de cada grupo de intervención

				Tamaño	Tamaño de	Foco de	Edad,	Masculino	IMC, media		Periodo de	Reversión	Otros
Autor	Año	Definición	País	de la	muestra		media		(DE)	Etnia %		del síndrome	
						intervención		%			seguimiento		resultados
									kg/m2				
				muestra	con MetS		(DE)					metabólico	
Anderssen, *et*	2007	IDF	Noruega	188	137	Estilo de	45 (2,5)	100	29	NR	1 año	Sí	-
*al*. [26]						vida (dieta							
						y ejercicio							
						supervisado)							
Athyros, *et al*.	2005	NCEP	Grecia	300	300	Atorvastina y	NR	63	31	NR	1 año	Sí	-
[27]						fenofibrato							
Bo, *et al*. [28]	2007	NCEP	Italia	335	239	Estilo de	56*	42*	30	NR	1 año	Sí	Incidencia
						vida (dieta							de T2DM
						y ejercicio							
						supervisado)							
Esposito, *et*	2004	NCEP	Italia	180	180	Estilo de vida	44	55	28	NR	2 años	Sí	-
*al*. [29]						(dieta)							
Esposito, *et*	2006	NCEP	Italia	100	100	Rosigilitazona	46 (4,5)	54	28	NR	1 año	Sí	-
*al*. [30]													
Geluk, *et al*.	2005	NCEP	Holanda	864	228	Pravastatina	55 (11)**	70**	29	96 %	4 años	Sí	Evento
[31]										Caucásico			CHD o
													muerte
Johnson, *et*		NCEP	Estados	334	69	Estilo de vida	53 (7)*	53*	30	NR	8 meses	Sí	-
*al*. [32]			Unidos			(ejercicio							
						supervisado)							
Orchard, *et al*.	2005	NCEP	Estados	3324	1711	Estilo de	51 (10,7)*	32*	34	55 %	3 años	Sí	-
[33]			Unidos			vida (dieta y				Blanco,			
						consejería				20 % Af-			
						deportiva)				Am, 16 %			
						Metformina				Hip, 9 %			
										otro			
Phelan, *et al*.	2007	NCEP	Estados	224	78	Estilo de	48 (9,9)*	37	38	80 %	1 año	Sí	-
[34]			Unidos			vida (dieta y				Blancos			
						consejería				18 % Af-			
						deportiva)				Am, 3 %			
						Sibutramina				Hisp.			
Ramachandran,	2006	OMS	India	521	233	Estilo de	46*	79*	26	Indio	3 años	Sí	Incidencia
*et al*. [35]						vida (dieta y				asiático			de T2DM
						consejería				nativo			
						deportiva)							
						Metformina							
Stewart, *et al*.	2005	NCEP	Estados	115	44	Estilo de	64 (5,7)*	49*	30	87 %	26 semanas	Sí	-
[36]			Unidos			vida (dieta				Blancos,			
						y ejercicio				11 % Af-			
						supervisado)				Am, 2 %,			
										otros			
van Gaal, *et*	2005	NCEP	Europa-	1507	564	Rimonabant	45*	21*	36	94 %	1 año	Sí	-
*al*. [37]			Estados							Blanco			
			Unidos										
Villareal, *et al*.	2006	NCEP	Estados	27	24	Estilo de	70*	33*	39	85 %	26 semanas	Sí	-
[38]			Unidos			vida (dieta				Blanco			
						y ejercicio							
						supervisado)							
Siu, *et al*. [25]	2015	NCEP	China	182	76	Estilo de vida	56 (9,1)	32	--	Asiático	1 año	Sí	-
						(ejercicio							
						supervisado)							
Yoo, *et al*. [24]	2012	NCEP	Corea	195	108	Estilo de vida	65 (3,38)	38	25.87(2.3)	Asiático	6 meses	Sí	-
						(educación, y ejercicio supervisado.							

DE: desviación estándar; Af-Am: afroamericano; IMC: índice de masa corporal; CHD: enfermedad coronaria; Hisp: hispano; IDF: International Diabetes Federation; NCEP: Programa Nacional de Educación sobre el Colesterol; NR, no reportado; T2DM: diabetes mellitus de tipo 2; OMS: Organización Mundial de la Salud * Los datos indicados corresponden a la prueba principal, no estaban disponibles para el subgrupo. ** Datos únicamente de edad, etc., incluida la diabetes mellitus. Fuente: Base de datos EMBASE y Medline, consultadas en octubre de 2017

Los contrastes más frecuentes (nueve artículos), se hicieron entre el estilo de vida y el grupo de control: Esposito, *et al*., 2004 ^29^; Stewart, *et al*., 2005 ^36^; Orchard, *et al*., 2005 ^33^; Villareal, *et al*., 2006 ^38^; Ramachandran, *et al*., 2006 ^35^; Anderssen, et al., 2007 ^26^; Bo, et al., 2007 ^28^; Yoo, *et al*., 2012 ^24^ y Siu, *et al*., 2015 ^25^. Además, en cinco artículos se compararon tratamientos con esquemas farmacológicos versus un grupo control: Orchard, et al., 2005 ^33^; van Gaal, et al., 2005 ^37^; Geluk, *et al*., 2005 ^31^; y Esposito, et al., 2006 ^30^, y Ramachandran, *et al*., 2006 ^35^.

Otro de los contrastes incluidos en los estudios fue entre la combinación de las intervenciones relacionadas con el estilo de vida y los tratamientos farmacológicos, la cual se registró en tres artículos: Orchard, *et al*., 2005 ^33^, Ramachandran, *et al*., 2006 ^35^ y Phelan, *et al*., 2007 ^34^.

Asimismo, en dos artículos se comparó la combinación de una intervención relacionada con el estilo de vida y el tratamiento farmacológico con cada una de estas intervenciones por separado: Ramachandran, *et al*., 2007 ^35^ y Phelan, *et al*., 2007 ^34^. Por último, solamente en un artículo se compararon la combinación del tratamiento farmacológico y la intervención relacionada con el estilo de vida, versus el grupo control. Cabe mencionar que más de un tipo de comparación puede aparecer en un solo artículo .

En el cuadro 1, se presentan también la categorización de cada grupo de intervención y el número de casos de reversión del síndrome metabólico por tipo de intervención. Al agregar los dos estudios publicados después del estudio de base ^12^, el metaanálisis incluyó los datos de 4.291 participantes con el síndrome, y los resultados que evidenciaron los beneficios de las intervenciones en el estilo de vida y las farmacológicas.

**Figura 3.**
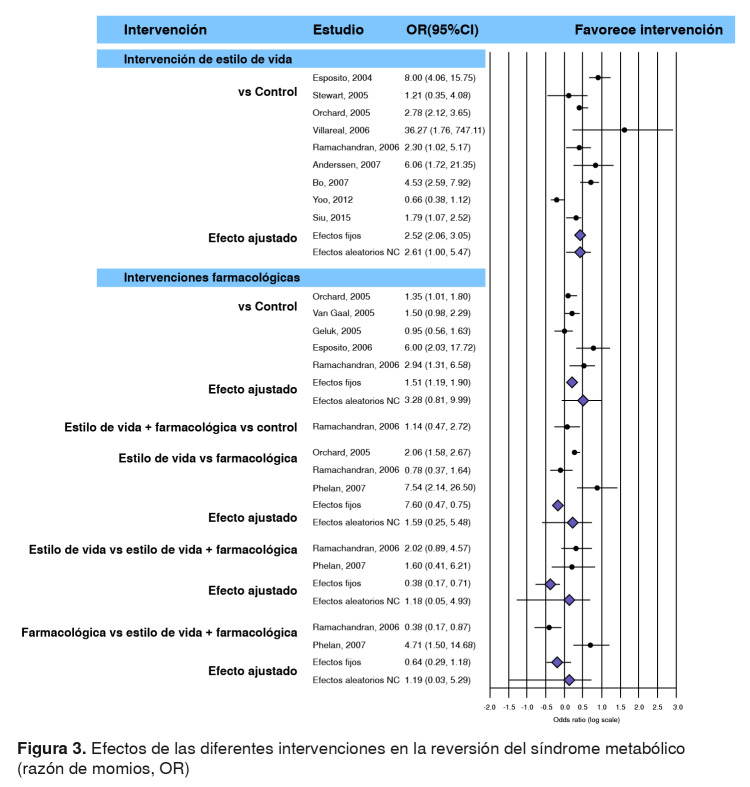
Efectos de las diferentes intervenciones en la reversión del síndrome metabólico (razón de momios, OR)

En la figura 3, se presenta el diagrama de bosque (forest plot) de los efectos de las diferentes intervenciones empleadas en los estudios analizados en la reversión del síndrome metabólico y el efecto combinado estimado con modelos de efectos fijos y efectos aleatorios no correlacionados. En ella, se aprecia también que, en contraste con los grupos de control, las intervenciones en el estilo de vida tienen más probabilidades de revertir el síndrome que las farmacológicas. Además, con respecto al modelo de efectos fijos, el efecto combinado de las intervenciones en el estilo de vida comparadas con los grupos de control es mayor (OR=2,52; IC95%=2,06-3,05) que el de las farmacológicas (OR=1,51; IC95%=1,19-1,90), así como en el caso de la combinación de ambas (OR=1,14; IC95%=0,47- 2,72). En cuanto al modelo de efectos aleatorios no correlacionados, el efecto combinado de las intervenciones en el estilo de vida se contrajo más (efectos aleatorios no correlacionados, OR=2,61; IC95%=1,00-5,47) que en las farmacológicas (efectos aleatorios no correlacionados, OR=3,39; IC95%=0,81- 9,99) en comparación con los grupos de control.

Al comparar directamente el efecto entre ambas intervenciones, las OR de reversión del síndrome metabólico al intervenir en el estilo de vida disminuyeron en 40 % en comparación con las intervenciones farmacológicas según los resultados del modelo de efectos fijos (OR=0,60; IC95%=0,47-0,75). Por otra parte, según el modelo de efectos aleatorios no correlacionados, la probabilidad de reversión mediante las intervenciones en el estilo de vida, fue 59 % mayor que la de las intervenciones farmacológicas.

En el cuadro 2, se presenta la comparación entre el método de efectos fijos y el método de efectos aleatorios no correlacionados, y las intervenciones comparadas con los tratamientos. Se observa que, en ambos modelos, las intervenciones relacionadas con el estilo de vida y la utilización de un fármaco obtuvieron OR mayores de 1 con respecto al grupo de control, lo que evidencia que son efectivas para revertir el diagnóstico de síndrome metabólico. Cabe destacar que el modelo de efectos aleatorios no correlacionados contradice lo obtenido con el de efectos fijos con respecto al cambio del estilo de vida en conjunto con un fármaco; este tratamiento conjunto fue beneficioso para revertir el síndrome al usar el modelo de efectos aleatorios no correlacionados y hacer la comparación con el grupo control.

**Cuadro 2:** Resultados de las comparaciones de tratamientos mediante modelos de efectos fijos y de efectos aleatorios no correlacionados

Comparación	Efectos fijos			Efectos aleatorios no correlacionados			
					
OR	(IC_95_%)		OR	(IC_95_%)		
	
Estilo de vida Vs. control	2,52	(2,06-3,05)	2,61		(1,00-	5,47)
Farmacológica Vs. control	1,51	(1,19-1,90)	3,39		(0,81-	9,99)
Farmacológica y estilo de vida Vs. control	0,96	(0,43-1,79)	2,31		(0,13-10,31)		
Farmacológica Vs. estilo de vida	0,60	(0,47-0,75)	1,59		(0,25-	5,48)
Farmacológica y estilo de vida Vs. estilo de vida	0,38	(0,17-0,71)	1,18		(0,05-	4,93)
Farmacológica + estilo de vida Vs. farmacológica	0,64	(0,29-1,18)	1,19		(0,03-	5,29)
DIC	167,57	125,74				

DIC: Deviation information criterion

Se utilizó el criterio de información de desviación (*deviance information criterion*) para elegir entre ambos modelos mediante la evaluación de la bondad de ajuste y se lo valoró como método de validación de los resultados. Como se observa en el cuadro 2, el modelo de efectos aleatorios presentó una mejor bondad de ajuste, debido a que el indicador de desviación para este modelo resultó ser menor (125,74) en comparación con el obtenido con el modelo de efectos fijos. Se fijó una distribución uniforme para configurar a priori un parámetro de la heterogeneidad de los estudios, por lo que se asumió que los errores de precisión de los estudios seguían un patrón uniforme.

En el cuadro 3, se presentan las OR de los modelos del artículo de referencia (metaanálisis directo y análisis de comparación mixta de tratamientos), comparadas con los modelos estimados (efectos fijos y efectos aleatorios no correlacionados), así como las estimaciones obtenidas al modelar un ajuste según la correlación entre las comparaciones, con lo que se consideraron las diferencias directas e indirectas para cada contraste requerido. Se observó que, en ambos casos, en el artículo de referencia, las intervenciones (estilo de vida, fármacos, y estilo de vida y fármacos en conjunto) presentaban ventajas mayores de 1, en comparación con el control, lo que confirmó el efecto positivo sobre la reversión del síndrome metabólico. Sin embargo, los intervalos de credibilidad para las intervenciones en el estilo de vida no incluyeron el 1, lo cual demostró la superioridad de este tipo de tratamiento comparado con los farmacológicos.

**Cuadro 3:** Comparación de los resultados del artículo de referencia con los de los actualizados

				Análisis de			Comparaciones			Efectos aleatorios	
Comparación	Metaanálisis directo			comparaciones mixtas			mixtas de tratamientos			no correlacionados	
				de tratamientos			actualizadas			actualizados	
	OR	(IC_95_%)		OR	(IC_95_%)		OR	(IC_95_%)		OR	(IC_95_%)
											
Estilo de vida Vs. control	3,81	(2,47 - 5,88)	4,43		(2,36-9,16)	6,06		(2,55-14,23)	2,606		(1 - 5,47)
Farmacológica Vs. control	1,59	(1,04 - 2,45)	1,73		(0,73-3,80)	3,13		(1,13 - 7,71)	3,387		(0,81 - 9,99)
Farmacológica y estilo de vida Vs. control	1,14	(0,48 - 2,72)	2,16		(0,63-8,29)	2.27		(0,26 - 8,93)	2,313		(0,13-10,31)
Farmacológica Vs. estilo de vida	0,49	(0,20 - 1,24)	0,39		(0,16-0,90)	0,58		(0,16 - 1,45)	1,586		(0,25 - 5,48)
Farmacológica y estilo de vida Vs. estilo de vida	0,53	(0,26 - 1,06)	0,49		(0,14-1,74)	0,41		(0,04 - 1,53)	1,177		(0,05 - 4,93)
Farmacológica y estilo de vida Vs. farmacológica	1,31	(0,11-15,13)	1,25		(0,36-4,49)	0,84		(0,08 - 3,16)	1,185		(0,03 - 5,29)

Nota: El modelo de efectos aleatorios no correlacionados se compara con el metaanálisis directo del artículo de Dunkley, *et al*., 2012

Por último, en el mismo cuadro 3, se reportan las OR de diez estudios resultantes del análisis de redes de evidencia y de las comparaciones mixtas de tratamientos a partir de modelos bayesianos. Se analizó la sensibilidad para valorar la bondad de ajuste del modelo completo en la estimación de las OR actualizadas, utilizando el método de comparaciones mixtas de tratamientos. El análisis consistió en excluir el estudio con los resultados más extremos en la efectividad, es decir, el de Villareal, *et al*. ^38^, en el cual el grupo de control no registró reversión alguna. El criterio de información de desviación del modelo en los diez estudios resultó en un valor de 126,64, pero al calcularlo eliminando los datos del estudio de Villareal, este indicador se elevó a 130,47.

## Discusión

Esta síntesis de la información permitió determinar el tipo de intervención más efectiva para revertir el síndrome. En la síntesis de Dunkley, *et al*. ^12^, los hallazgos no fueron suficientes para demostrar las diferencias entre los tratamientos farmacéuticos y las intervenciones en el estilo de vida; sin embargo, al añadir los estudios publicados en el 2012 por Yoo, et al. ^24^ y Siu, *et al*. ^25^, se encontró una efectividad significativa de las estrategias basadas en el ejercicio, la dieta y la educación.

Con base en los datos recopilados sobre la red de tratamientos relacionados con la posibilidad de reversión, se pudo trazar una trayectoria histórica en torno a los datos disponibles. Hay claridad en cuanto a que ha habido un cambio en la manera de reportar el producto de la estimación reportada para la efectividad de las intervenciones y sus efectos en personas con síndrome metabólico. Se encontraron solo dos estudios nuevos con un reporte binario del síndrome. Se excluyeron los estudios que involucraban una jerarquía mucho más compleja, al considerar los efectos en los diferentes componentes del síndrome, pues transformaban la variable de respuesta de una distribución binomial a una distribución multivariada, con una amplia gama de categorías posibles en las que se localizarían los individuos que cumplen con los criterios de clasificación del síndrome metabólico.

La comparación de los resultados de la red de tratamientos presentada en la figura 2 con la estimación actualizada de las OR mediante comparaciones mixtas de tratamientos, permitió valorar un acervo de información mucho más abundante, estimar las medidas con intervalos de credibilidad mucho más sólidos y observar de manera más detallada el tipo de patrón que se construye conforme se agrega más información relevante.

El contraste con mayor información se obtiene al comparar el estilo de vida con algún otro factor basal que sirva para conformar un grupo de control y favorezca la comparación entre los tratamientos. En este caso, se encontraron nueve estimaciones directas en los estudios analizados, después de excluir los artículos que no reportaron ningún control.

Al integrar la información indirecta de esta comparación, la probabilidad bayesiana de revertir el síndrome fue seis veces mayor cuando se involucró a las personas en procesos de aprendizaje general sobre su nutrición y la forma de ejercitarse. El elemento de comparación generalizado fueron las intervenciones de base en las que se les dio a los participantes algún consejo general sobre hábitos saludables o se les dijo que continuaran con los que tenían en el momento del estudio.

También se demuestra que las farmacoterapias tienen un efecto importante en la reversión del síndrome; esta comparación fue la segunda más informada, con cinco comparaciones directas obtenidas de la información utilizada. Sin embargo, en este caso, la intensidad del efecto de la intervención farmacéutica se reduce en 50 % con respecto a la intervención en el estilo de vida. Por lo tanto, el tratamiento basado en fármacos ofrece una efectividad tres veces mayor de revertir un diagnóstico del síndrome, considerando una variabilidad de la OR entre 1,13 y 7,71. Esto podría estar vinculado con el hecho de que no necesariamente se opta por los tratamientos farmacológicos para el manejo de los componentes del síndrome metabólico.

En cuanto a las estimaciones de la comparación basal de la intervención combinada, se encontró un efecto elevado bastante apreciable; sin embargo, dado que esta comparación fue la menos reportada, con una sola comparación directa, el intervalo de credibilidad incluyó la unidad. Por lo tanto, sería necesario agregar más información para valorar si la relación se mantiene en una efectividad más dispersa o si se confirma la necesidad de descartarla.

Ahora bien, esta es una comparación que, en la práctica clínica, siempre se va a dar en ciertos grupos de individuos con condiciones que podrían requerir medicación, como la hipertensión arterial sistémica. Es importante anotar que esta intervención debería estudiarse dadas las posibles interacciones. Las demás comparaciones no resultaron significativas, ya que, a pesar de tener más datos, los intervalos mantuvieron la amplitud que incluye la unidad, o de efecto nulo.

En cuanto a las limitaciones de este estudio, está la necesidad de establecer una red de tratamientos mucho más específica y explorar los detalles de las intervenciones, para posibilitar diseños innovadores en la gestión de las políticas de salud pública. Además, debería hacerse un análisis de sesgo de publicación, con el fin de observar si estas estimaciones merecen un nuevo ajuste.

En conclusión, al comparar los resultados de estos 15 artículos con el de Dunkley, *et al*., el cual incluyó 13 estudios ^12^, se demostró que, en ambos casos, el uso de cualquiera de las intervenciones o tratamientos ayudó a revertir el síndrome metabólico. Sin embargo, la actualización permite demostrar una superioridad en la efectividad de las intervenciones por estilo de vida.
